# Loco-regional recurrence trend and prognosis in young women with breast cancer according to molecular subtypes: analysis of 1099 cases

**DOI:** 10.1186/s12957-021-02214-5

**Published:** 2021-04-13

**Authors:** Yang Li, Su Lu, Yuhan Zhang, Shuaibing Wang, Hong Liu

**Affiliations:** 1 The Second Surgical Department of Breast Cancer, Tianjin Medical University Cancer Institute and Hospital, National Clinical Research Center for Cancer & Key Laboratory of Cancer Prevention and Therapy, Tianjin’s Clinical Research Center for Cancer, Tianjin, 300060 China; 2 Oncology Department, Taikang Xianlin Drum Tower Hospital, School of Medicine, Nanjing University, Nanjing, 210046 Jiangsu Province China; 3 Oncology Department, Hebei PetroChina Central Hospital, Langfang, 065000 Hebei Province China

**Keywords:** Young breast cancer, Local recurrence, Regional recurrence, Distant metastases, Molecular subtype, Overall survival

## Abstract

**Background:**

The number of young patients diagnosed with breast cancer is on the rise. We studied the rate trend of local recurrence (LR) and regional recurrence (RR) in young breast cancer (YBC) patients and outcomes among these patients based on molecular subtypes.

**Methods:**

A retrospective cohort study was conducted based on data from Tianjin Medical University Cancer Institute and Hospital for patients ≤ 35 years of age with pathologically confirmed primary invasive breast cancer surgically treated between 2006 and 2014. Patients were categorized according to molecular subtypes on the basis of hormone receptor (HR) and human epidermal growth factor receptor 2 (HER2) status. The 5-year rates for LR, RR, and distant metastases (DM) were estimated by Kaplan-Meir statistics. Nelson-Aalen cumulative-hazard plots were used to describe local recurrence- and distant metastasis-free intervals.

**Results:**

We identified 25,284 patients with a median follow-up of 82 months, of whom 1099 (4.3%) were YBC patients ≤ 35 years of age. The overall 5-year LR, RR, and DM rates in YBC patients were 6.7%, 5.1%, and 16.6%, respectively. The LR and RR rates demonstrated a decreasing trend over time (*P* = 0.028 and *P* = 0.015, respectively). We found that early-stage breast cancer and less lymph node metastases increased over time (*P* = 0.004 and *P* = 0.007, respectively). Patients with HR−/HER2+ status had a significantly higher LR (HR 20.4; 95% CI, 11.8–35.4) and DM (HR 37.2; 95% CI, 24.6–56.3) at 10 years. Breast-conserving surgery (BCS) or mastectomy did not influence rates of LR and RR. In the overall population, the 5-year survival of YBC patients exceeded 90%.

**Conclusions:**

The rates of LR and RR with YBC patients demonstrated a downward trend and the proportion of early-stage breast cancer increased between 2006 and 2014. We report the highest LR rates in this young population were associated with HR−/HER2+ tumors.

## Introduction

It has been estimated that 4% of females < 40 years of age were diagnosed with breast cancer in the United States in 2017 and breast cancer is the leading cause of cancer deaths among women 20–59 years of age [1, 2]. Prior studies have revealed that young age is a known risk prognostic factor for breast cancer patients [3–8]. This finding is reflected by larger tumors, higher grade, advanced stage, more lymph node metastases, a higher prevalence of human epidermal growth factor receptor (HER)2 over-expression, and estrogen receptor (ER) negativity in young women with breast cancer [4, 5, 7]. With respect to detrimental gene expression, Azim et al. reported higher expression of gene signatures related to proliferation, stem cells, and endocrine resistance in tumors associated with young age [9]. In addition, higher expression of epithelial growth factor receptor (EGFR) mRNA, which BRCA1/2-associated breast tumors overexpress [10], is a significant predictor of poor prognosis in young women [5].

The relative risk of loco-regional recurrence (LRR) increases by 7% for every year of decrease in age [11]. Previous research showed that young women with breast cancer who undergo breast-conserving surgery (BCS) have higher rates of developing LRR compared with women who undergo mastectomy, but the overall survival (OS) is not affected [12, 13]. Despite higher rates of LRR in young patients, several studies had shown a declining trend in the LRR rate over the past two decades [14–16]. In addition, there has been a significant decline in the occurrence of distant metastases (DM) [17, 18] and increase in the OS over the last years in YBC patients owing to the evolution of improved adjuvant systemic treatment and raising consciousness of physical examination [19, 20].

Breast cancer arising in young women is more likely to develop into more aggressive tumor subtypes, including a greater proportion of triple-negative and HER2 over-expressing subtypes [5, 6, 8]. Accumulating evidence has demonstrated a strong relationship between molecular subtypes and prognosis in YBC patients [21–32]. A number of reports have shown a worse OS rate in young women with luminal B breast cancer [27, 28, 31, 32], whereas other research has suggested that triple-negative and HER2 over-expressing tumors are strong predictors of disease recurrence [29, 30, 33]. Therefore, larger, well-designed prospective clinical studies are needed to explore this relationship.

The trend in LRR rates in YBC patients in recent years has not been established. We therefore evaluated the trend in LRR and determine the impact of molecular subtypes on LRR and OS in young women diagnosed with breast cancer.

## Methods

### Patients

This was a retrospective study that included breast cancer patients ≤ 35 years of age at Tianjin Medical University Cancer Institute and Hospital from January 2006 to December 2014. There were 25,284 patients diagnosed with breast cancer in our hospital during the 9-year period, of whom 1307 were ≤ 35 years of age. Patients < 35 years of age with pathologically confirmed primary invasive breast cancer and underwent surgery from 2006 to 2014 were selected for our study. Subjects with non-invasive cancer (54 cases), including ductal carcinoma in situ (16 cases), primary metastatic breast cancer (20 cases), and primary bilateral breast cancer (42 cases), were excluded. We also excluded patients who did not have electronic medical records in our institution and who could not be contacted by telephone or mail to confirm survival status (92 cases). A total of 1099 young women with breast cancer met the inclusion criteria for our study.

#### Clinicopathological information of patients

We collected the following patient demographics: age; family history of breast cancer; reproductive history; and breastfeeding history. The tumor characteristics included tumor size, stage, lymph node status, histologic grade, and pathologic type. We classified cancer into five molecular subtypes according to hormone receptor (HR) and HER2 status, as follows: HR-positive/Her2-negative; HR-positive/Her2-positive; HR-negative/Her2-positive; HR-negative/Her2-negative; and unknown. Molecular subtype was defined by immunohistochemical staining features of HR (estrogen receptor [ER] and/or progesterone receptor [PR]) and HER2. Categorization based on staining features was as follows: ER and PR staining < 1% was defined as negative; ER and/ or PR staining ≥ 1% was defined as positive [34]; HER2 0/1 was defined as negative; and HER2 2+ was defined as negative or positive by fluorescence in situ hybridization (FISH) and positive by HER2 3+. Information regarding adjuvant chemotherapy, radiotherapy, hormonal therapy, ovarian function suppression, and trastuzumab therapy were obtained from the hospital and follow-up records.

### Variable definitions

The follow-up data for LR, RR, DM, and OS were abstracted from the electronic medical records, paper medical documents, telephone, and mail. For patients at the time of contact had died, available family members provided the requested information. Follow-up started on the day of surgery to the date of any type of recurrence, death, the last contact according to the medical record, or in-person contact. LR was defined as recurrence of ipsilateral breast cancer after BCS or chest wall recurrence after mastectomy. RR referred to the occurrence of tumor in the ipsilateral regional lymph nodes, including the axillary, infra- or supra-clavicular or internal mammary lymph nodes. DM was defined as recurrence beyond LR and RR. We defined OS as the time from surgery to death from any cause or last follow-up. The local recurrence-free interval (LRFI) was defined as the interval from surgery to local recurrence or the date of last follow-up.

### Statistical analysis

Descriptive statistics were performed to examine the demographic characteristics of young patients surgically treated between 1 January 2006 and 31 December 2014. The percentage of clinicopathologic and therapeutic regimen among YBC patients were compared for the different molecular subtypes using a chi-square test. Moreover, tumor characteristics for all YBC patients according to the time of diagnosis were assessed over time.

We used Kaplan-Meier survival estimates to calculate overall 5-year LR, RR, and DM rates for the young patients with breast cancer and the trends of LR, RR and DM over time were assessed by using linear regression analysis. Moreover, LR, RR, and DM rates of YBC patients treated between 2006 and 2014 according to various molecular subtypes were calculated. We performed univariate and multivariate Cox proportional hazard model to examine the influence of different variables on LR, RR, and DM. Hazard ratios and the associated 95% confidence intervals (CIs) were obtained based on Cox regression analysis. The OS was summarized by Kaplan-Meier survival curves according to tumor subtypes and compared using log-rank test univariate analyses. Nelson-Aalen cumulative-hazard plots were used to describe the LRFI and distant metastases-free interval (DMFI). Subsequently, 5- and 10-year estimates of LRFI, regional recurrence-free interval (RRFI), DMFI, and OS according to various molecular subtypes were calculated using Kaplan-Meier survival analysis.

*P* values < 0.05 were considered statistically significant and all tests were two-tailed. Analyses were performed using SPSS 22.0 and STATA software 14.1.

## Results

### Patient characteristics

A total of 1099 YBC patients who were surgically treated were enrolled in our study from 2006 to 2014. This cohort accounted for 4.3% of the total population of patients who were diagnosed with breast cancer in our hospital during the 9-year period (*n* = 25,284). The median follow-up time was 82 months. The demographic characteristics of the YBC patients are shown in Table 1. Seventy-five percent of the patients had early-stage breast cancer (stages I and II). Among the patients, 54.0%, 10.9%, 6.8%, and 18.3% of patients were HR+/HRE2−, HR+/Her2+, HR−/Her2+, and HR−/HER2− subtypes, respectively. The baseline clinicopathologic and treatment characteristics differed by tumor subtype, as shown in Table 2. Patients with HER2 2+ status who were not subsequently detected by FISH were classified as unknown subtype. HR+/HER2− tumors tended to be smaller in size (*P* = 0.007), lower stage (*P* < 0.001), and lower histologic grade (*P* < 0.001) compared with the other subtypes. Patients with HR−/HER2+ status were likely to have larger tumors (*P* = 0.007) and patients with HER2-negative breast cancer presented with fewer lymph node metastases, while HER2-positive tumors tended to have > 9 lymph node metastases (*P* < 0.001).

**Table 1:** Demographic characteristics of young patients surgically treated between January 1, 2006, and December 31, 2014 (*n* = 1099)

Characteristics	No. of patients (%)
BC family history	
Yes	113 (10.3)
No	969 (88.2)
Unknown	17 (1.5)
Reproductive history*	
Yes	827 (75.3)
No	257 (23.4)
Unknown	15 (1.4)
Breastfeeding history	
Yes	745 (67.8)
No	337 (30.7)
Unknown	17 (1.5)
Tumor size	
T1	420 (38.2)
T2	508 (46.2)
T3	88 (8.0)
T4	16 (1.5)
Tx	67 (6.1)
Stage	
I	282(25.7)
II a	383 (34.8)
II b	159 (14.5)
III a	126 (11.5)
III b	8 (0.7)
III c	97 (8.8)
Unknown	44 (4.0)
Lymph node metastasis	
N0	585 (53.2)
N1	276(25.1)
N2	124 (11.3)
N3	94 (8.6)
Unknown	20 (1.8)
Histological grade	
Well differentiated	35 (3.2)
Moderately differentiated	594 (54.0)
Poorly differentiated	163 (14.8)
Unknown	307 (27.9)
Pathological type	
Invasive ductal carcinoma	999 (90.9)
Invasive lobular carcinoma	10 (0.9)
Others	90 (8.2)
Final surgery	
Breast-conserving surgery	257 (23.4)
Mastectomy	842 (76.6)
Biomarker subtype	
HR+/HER2−	594 (54.0)
HR+/HER2+	120 (10.9)
HR−/HER2+	75 (6.8)
HR−/HER2−	201 (18.3)
Unknown	109 (9.9)
Neoadjuvant chemotherapy	
Yes	176 (16.0)
No	923 (84.0)
Adjuvant chemotherapy regimens	
Anthracycline-based	140 (12.7)
Anthracycline- and taxane-based	884 (80.4)
Unknown	67 (6.1)
None	8 (0.7)
Radiotherapy	
Yes	557 (50.7)
No	453 (41.2)
Unknown	89 (8.1)
Endocrine therapy	
Yes	589 (53.6)
No	316 (28.8)
Unknown	194 (17.7)
Ovarian function suppression	
Yes	187 (17.0)
No	545 (49.6)
Unknown	367 (33.4)
Trastuzumab treatment	
Yes	85 (7.7)
No	836 (76.1)
Unknown	178 (16.2)

*BC*, breast cancer; *HR+*, hormone receptor positive, *HR−* hormone receptor negative, *HER2+* human epidermal growth factor 2 positive, *HER2−* human epidermal growth factor 2 negative

*Reproductive history: Yes means they had children

**Table 2:** Baseline demographic characteristics of all young patients according to various molecular subtypes (*n* = 1099)

Characteristics	HR+/HER2− (*n* = 594)	HR+/HER2+ (*n* = 120)	HR−/HER2+ (*n* = 75)	HR−/HER2− (*n* = 201)	Unknown (*n* = 109)	*P*
BC family history						0.023
YES	62 (10.4)	16 (13.3)	5 (6.7)	21 (10.4)	9 (8.3)	
NO	524 (88.2)	104 (86.7)	68 (90.7)	179 (89.1)	94 (86.2)	
Unknown	8 (1.3)	0 (0)	2 (2.7)	1 (0.5)	6 (5.5)	
Reproductive history						0.003
YES	451 (75.9)	92 (76.7)	61 (81.3)	146 (72.6)	77 (70.6)	
NO	136 (22.9)	28 (23.3)	12 (16.0)	55 (27.4)	26 (23.9)	
Unknown	7 (1.2)	0 (0)	2 (2.7)	0 (0)	6 (5.5)	
Breastfeeding history						< 0.001
YES	410 (69.0)	79 (65.8)	59 (78.7)	128 (63.7)	69 (63.3)	
NO	177 (29.8)	41 (34.2)	14 (18.7)	72 (35.8)	33 (30.3)	
Unknown	7 (1.2)	0 (0)	2 (2.7)	1 (0.5)	7 (6.4)	
Tumor size						0.007
T1	250 (42.1)	39 (32.5)	17 (22.7)	69 (34.3)	45 (41.3)	
T2	261 (43.9)	61 (50.8)	40 (53.3)	103 (51.2)	43 (39.4)	
T3	45 (7.6)	10 (8.3)	8 (10.7)	12 (6.0)	13 (11.9)	
T4	3 (0.5)	5 (4.2)	3 (4.0)	4 (2.0)	1 (0.9)	
Tx	35 (5.9)	5 (4.2)	7 (9.3)	13 (6.5)	7 (6.4)	
Stage						< 0.001
I	166 (27.9)	27 (22.5)	8 (10.7)	48 (23.9)	33 (30.3)	
II a	199 (33.5)	40 (33.3)	26 (34.7)	90 (44.8)	28 (25.7)	
II b	85 (14.3)	16 (13.3)	13 (17.3)	28 (13.9)	17 (15.6)	
III a	72 (12.1)	15 (12.5)	12 (16.0)	17 (8.5)	10 (9.2)	
III b	2 (0.3)	1 (0.8)	2 (2.7)	3 (1.5)	0 (0)	
III c	48 (8.1)	17 (14.2)	12 (6.0)	4 (2.0)	16 (14.7)	
Unknown	22 (3.7)	4 (3.3)	2 (2.7)	11 (5.5)	5 (4.6)	
LN metastasis						< 0.001
N0	312 (52.5)	55 (45.8)	36 (48.0)	134 (66.7)	48 (44.0)	
N1	161 (27.1)	30 (25.0)	14 (18.7)	46 (22.9)	25 (22.9)	
N2	70 (11.8)	16 (13.3)	13 (17.2)	16 (8.0)	9 (8.3)	
N3	46 (7.7)	17 (14.2)	12 (16.0)	2 (1.0)	17 (15.6)	
Unknown	5 (0.8)	2 (1.7)	0 (0)	3 (1.5)	10 (9.2)	
Histological grade						< 0.001
Well	29 (4.9)	0 (0)	1 (1.3)	2 (1.0)	3 (2.8)	
Moderately	371 (62.5)	67 (55.8)	36 (48.0)	78 (38.8)	42 (38.5)	
Poorly	50 (8.4)	25 (20.8)	16 (21.3)	52 (25.9)	20 (18.3)	
Unknown	144 (24.2)	28 (23.3)	22 (29.3)	69 (34.3)	44 (40.4)	
Pathological type						0.906
IDC	534 (89.9)	110 (91.7)	71 (94.7)	183 (91.0)	101 (92.7)	
IBC	7 (1.2)	1 (0.8)	0 (0)	1 (0.5)	1 (0.9)	
Others	53 (8.9)	9 (7.5)	4 ( (5.3)	17 (8.5)	7 (6.4)	
Final surgery						0.002
BCS	143 (24.1)	19 (15.8)	14 (18.7)	64 (31.8)	17 (15.6)	
Mastectomy	451 (75.9)	101 (84.2)	61 (81.3)	137 (68.2)	92 (84.4)	
NACT						0.003
YES	80 (13.5)	19 (15.8)	23 (30.7)	33 (16.4)	21 (19.3)	
NO	514 (86.5)	101 (84.2)	52 (69.3)	168 (83.6)	88 (80.7)	
ACT						0.046
A-based	72 (12.1)	17 (14.2)	12 (16.0)	24 (11.9)	15 (13.8)	
A- and T-based	490 ( (82.5)	97 (80.8)	58 (77.3)	162 (80.6)	77 (70.6)	
Unknown	28 (4.7)	6 (5.0)	5 (6.7)	12 (6.0)	16 (14.7)	
None	4 (0.7)	0 (0)	0 (0)	3 (1.5)	1 (0.9)	
Radiotherapy						< 0.001
YES	304 (51.2)	59 (49.2)	46 (61.3)	105 (52.2)	43 (39.4)	
NO	254 (42.8)	52 (43.3)	24 (32.0)	85 (42.3)	38 (34.9)	
Unknown	36 (6.1)	9 (7.5)	5 (6.7)	11 (5.5)	28 (25.7)	
ET						< 0.001
YES	467 (78.6)	89 (74.2)	0 (0)	0 (0)	33 (30.3)	
NO	27 (4.5)	8 (6.7)	70 (93.3)	194 (96.5)	17 (15.6)	
Unknown	100 (16.8)	23 (19.2)	5 (6.7)	7 (3.5)	59 (54.1)	
OFS						< 0.001
YES	147 (24.7)	34 (28.3)	0 (0)	0 (0)	6 (5.5)	
NO	215 (36.2)	46 (38.3)	67 (89.3)	189 (94.0)	28 (25.7)	
Unknown	232 (39.1)	40 (33.3)	8 (10.7)	12 (6.0)	75 (68.8)	
TT						< 0.001
YES	2 (0.3)	51 (42.5)	32 (42.7)	0 (0)	0 (0)	
NO	553 (93.1)	41 (34.2)	23 (30.7)	195 (97.0)	24 (22.0)	
Unknown	39 (6.6)	28 (23.3)	20 (26.7)	6 (3.0)	85 (78.0)	

All data are given as No. of patients (%). None represents no chemotherapy has been adopted

*LN*, lymph node; *IDC*, invasive ductal carcinoma; *IBC*, invasive lobular carcinoma; *NACT*, neoadjuvant chemotherapy; *BCS*, breast conserving surgery; *A-based*, anthracycline-based; *A- and T-based*, anthracycline- and taxane-based; *OFS*, ovarian function suppression; *ET*, endocrine therapy; *TT*, trastuzumab therapy

As for treatment, patients with HER2-negative tumors generally underwent BCS (*P* = 0.002) compared with HER2-positive tumors. The percentage of patients with HR−/HER2+ and HR−/HER2− statuses receiving neoadjuvant chemotherapy (*P* = 0.003) and radiotherapy treatment (*P* < 0.001) was higher than other subtypes. No statistically significant difference was observed in chemotherapy among patients with different molecular subtypes. There were 194 women with HER2-positive status and 83 patients received trastuzumab therapy, of whom 20 relapsed after surgery in the current study.

**Fig. 1:**
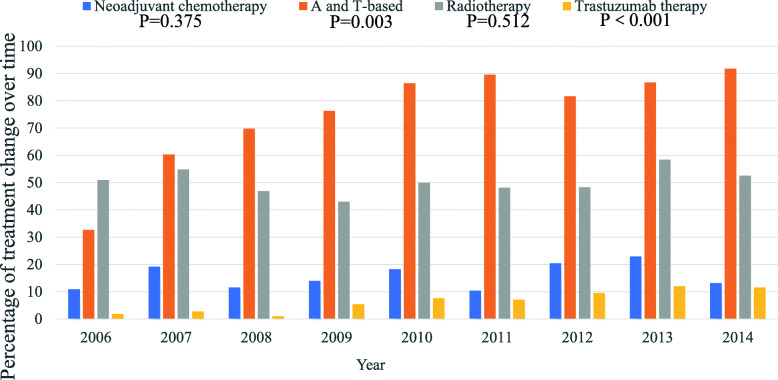
Time trends of treatment modalities in YBC patients during the study period of 2006 to 2014. *P* values for time trends of different treatment modalities were evaluated by linear regression analyses

In addition, we studied the distribution of tumor characteristics and treatment for all YBC patients over time shown in Table 3. Tumor size, histologic grade, pathologic type, and type of surgery did not vary significantly between 2006 and 2014. Of note, there were distinct proportional shifts of stage and lymph node metastases over time (*P* = 0.004 and *P* = 0.007, respectively). The proportion of N1 increased (*P* = 0.016 using linear regression analyses), while N2 and N3 showed a declining trend, although no significant difference was detected using linear regression analyses. The percentage of patients with stage II breast cancer was higher and the percentage of patients with breast cancer stage III trended down over time. The proportion of patients receiving neoadjuvant chemotherapy (*P* = 0.375) and radiotherapy (*P* = 0.512) did not increase significantly over time (Fig. 1). There was a significant proportional shift of receiving chemotherapy during the study period: the proportion of patients treated with anthracycline- and taxane-based regimen increased (*P* = 0.003). The proportion of patients receiving trastuzumab increased over time (*P* < 0.001).

**Table 3:** Tumor characteristics for all young breast cancer patients according to the time of diagnosis (*n* = 1099)

	2006	2007	2008	2009	2010	2011	2012	2013	2014	*P*
	*n* = 55	*n* = 73	*n* = 96	*n* = 93	*n* = 132	*n* = 154	*n* = 147	*n* = 166	*n* = 183	
Tumor size										
T1	21 (38)	27 (37)	39 (41)	37 (40)	45 (34)	64 (42)	49 (33)	62 (37)	76 (42)	0.539
T2	30 (55)	34 (47)	43 (45)	42 (45)	61 (46)	65 (42)	75 (51)	82 (49)	76 (42)	
T3	2 (4)	3 (4)	5 (5)	5 (5)	16 (12)	13 (8)	15 (10)	12 (7)	17 (9)	
T4	1 (2)	2 (3)	3 (3)	0 (0)	1 (1)	1 (1)	4 (3)	2 (1)	2 (1)	
Tx	1 (2)	7 (10)	6 (6)	9 (10)	9 (7)	11 (7)	4 (3)	8 (5)	12 (7)	
Stage										0.004
I	11 (20)	21 (29)	27 (28)	24 (26)	30 (23)	40 (26)	35 (24)	45 (27)	49 (27)	
II a	24 (44)	20 (27)	37 (39)	36 (39)	41 (31)	47 (31)	56 (38)	51 (31)	71 (39)	
II b	4 (7)	11 (15)	9 (9)	13 (14)	17 (13)	19 (12)	27 (18)	36 (22)	23 (13)	
III a	13 (23)	7 (10)	8 (8)	8 (9)	22 (17)	20 (13)	13 (9)	14 (8)	21 (12)	
III b	0 (0)	2 (3)	0 (0)	0 (0)	0 (0)	0 (0)	2 (1)	2 (1)	2 (1)	
III c	3 (6)	5 (7)	14 (15)	4 (4)	14 (11)	21 (14)	12 (8)	13 (8)	11 (6)	
Unknown	0 (0)	7 (10)	1 (1)	8 (9)	8 (6)	7 (5)	2 (1)	5 (3)	6 (3)	
LNM										0.007
N0	30 (55)	42 (58)	56 (58)	52 (56)	67 (51)	73 (47)	80 (54)	83 (50)	102 (56)	
N1	8 (15)	15 (21)	19 (20)	26 (28)	30 (23)	38 (25)	41 (28)	54 (33)	45 (25)	
N2	13 (24)	11 (15)	6 (6)	10 (11)	19 (14)	20 (13)	12 (8)	15 (9)	18 (10)	
N3	4 (7)	5 (7)	14 (15)	5 (5)	14 (11)	21 (14)	11 (8)	11 (7)	9 (5)	
Unknown	0 (0)	0 (0)	1 (1)	0 (0)	2 (2)	2 (1)	3 (2)	3 (2)	9 (4.9)	
HG										0.415
Well	4 (7)	2 (3)	6 (6)	3 (3)	4 (3)	8 (5)	3 (2)	3 (2)	2 (1)	
Moderately	32 (58)	35 (48)	47 (49)	53 (57)	76 (58)	84 (55)	86 (59)	87 (52)	94 (51)	
Poorly	6 (11)	10 (14)	11 (12)	10 (11)	16 (12)	26 (17)	23 (16)	29 (18)	32 (18)	
Unknown	13 (24)	26 (36)	32 (33)	27 (30)	36 (27)	36 (23)	35 (24)	47 (28)	55 (30)	
PT										0.620
IDC	48 (87)	68 (93)	80 (83)	86 (93)	119 (90)	140 (90)	136 (93)	153 (92)	169 (92)	
ILC	0 (0)	1 (1)	1 (1)	1 (1)	2 (2)	2 (1)	0 (0)	1 (1)	2 (1)	
Others	7 (13)	4 (6)	15 (16)	6 (7)	12 (8)	12 (8)	11 (8)	12 (7)	12 (7)	
MS										0.043
HR+/HER2−	32 (58)	49 (67)	51 (53)	50 (54)	68 (52)	90 (58)	72 (49)	89 (54)	93 (51)	
HR+/HER2+	9 (16)	9 (12)	7 (7)	5 (5)	9 (7)	21 (14)	15 (10)	22 (13)	23 (13)	
HR−/HER2+	3 (6)	6 (8)	6 (6)	5 (5)	12 (9)	13 (8)	12 (8)	11 (7)	7 (4)	
HR−/HER2−	8 (15)	6 (8)	20 (21)	26 (28)	33 (25)	16 (10)	28 (19)	27 (16)	37 (20)	
Unknown	3 (6)	3 (4)	12 (13)	7 (8)	10 (8)	14 (9)	20 (14)	17 (10.2)	23 (13)	
Final surgery										0.792
BCS	15 (27)	22 (30)	23 (24)	17 (18)	30 (23)	32 (21)	34 (23)	42 (25)	42 (23)	
Mastectomy	40 (72)	51 (70)	73 (76)	76 (82)	102 (77)	122 (79)	113 (77)	124 (75)	141 (77)	

All data are given as no. of patients (%). Percentages may not add up to 100% as a result of rounding

*LNM*, lymph node metastasis; *HG*, histological grade; *PT*, pathological type; *IDC*, invasive ductal carcinoma; *ILC*, invasive lobular carcinoma; *NACT*, neoadjuvant chemotherapy; *MS*, molecular subtype; *BCS*, breast conserving surgery

### Recurrence rates

LR occurred in 83 patients in this study. There were 11 cases receiving surgical resection only, 35 cases using surgery and chemotherapy, 20 patients receiving chemotherapy only, 12 cases taking surgery and radiotherapy and chemotherapy, and the remaining 5 cases were unknown. A total of 211 patients occurred DM. The treatment of 34 patients after recurrence was unknown and the remaining 177 patients were treated with chemotherapy as the main combination therapy. The overall 5-year LR, RR, and DM rates in YBC patients were 6.7%, 5.1%, and 16.6%, respectively. We used linear regression analyses to evaluate the time trend of recurrence rates over the 9-year period. The LR and RR rates demonstrated a decreasing trend over time (*P* = 0.028 and *P* = 0.015, respectively). The DM rate also declined, although the difference was not statistically significant (*P* = 0.228), as is shown in Table 4.

**Table 4:** 5-year LR, RR, and DM rates of young breast cancer patients treated between 2006 and 2014

Year	No. of patients	LR, no. (%)	*P*	RR, no. (%)	*P*	DM, no. (%)	*P*
Overall	1099	69(6.7)		52(5.1)		176(16.6)	
2006	55	7(13.0)	0.028	4(7.4)	0.015	10(18.2)	0.228
2007	73	5(7.3)		10(14.4)		14(19.4)	
2008	96	12(13)		9(9.8)		17(18.3)	
2009	93	4(4.5)		3(3.3)		16(17.2)	
2010	132	7(5.6)		9(7.1)		17(13.1)	
2011	154	12(8.2)		7(4.7)		32(21.3)	
2012	147	8(6.1)		2(1.5)		25(17.6)	
2013	166	8(4.9)		5(3.2)		20(12.3)	
2014	182	6(3.4)		3(1.7)		25(15.8)	

*LR*, local recurrence; *RR*, regional recurrence; *DM*, distant metastasis

**Fig. 2:**
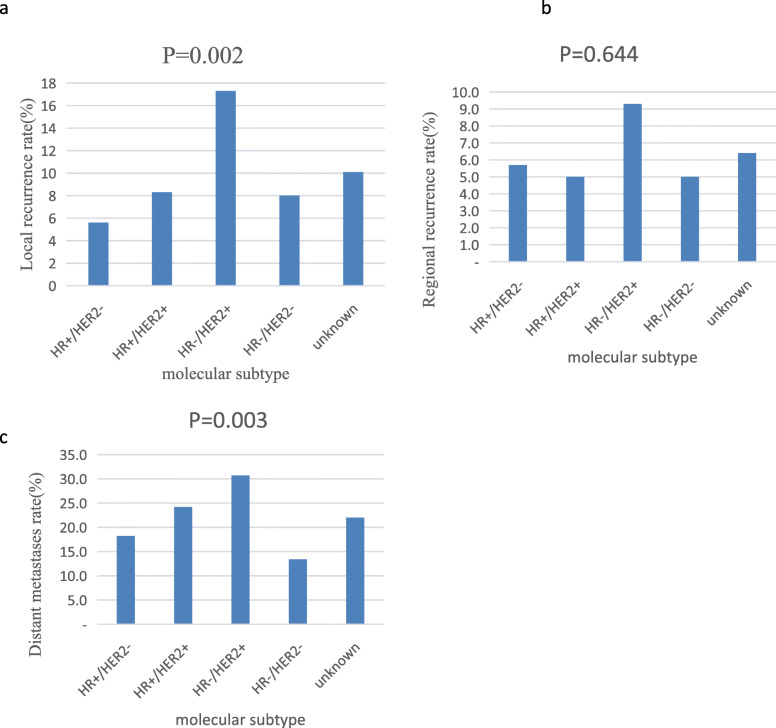
LR (**a**), RR (**b**), and DM (**c**) rates of young breast cancer patients treated between 2006 and 2014 according to various biomarker subtypes (*n* = 1099)

There were statistically significant differences in the LR and DM rates in patients with various tumor subtypes (*P* = 0.002 and *P* = 0.003, respectively; Fig. 2). Patients with HR−/HER2+ tumors had the highest recurrence rate compared with the other subtypes (LR: 17.3%, RR: 9.3%, and DM: 30.7%). Patients with HR+/HER2− status displayed the lowest LR rate (5.6%), whereas the triple-negative subtype showed the lowest DM rate (13.4%). We used univariate and multivariate Cox proportional hazard models to analyze the prognostic factors, as shown in Table 5 and Table 6. Patients with larger tumors and more lymph node metastases had increased HR in multivariate analyses for LRR (*P* < 0.01). The type of surgery did not influence the risk of LR and RR. LR and RR were 17.0% and 5.4% after BCS versus 7.9% and 8.3% after a mastectomy (*P* = 0.124, *P* = 0.296, respective). In addition, a total of 36 patients underwent sentinel lymph node biopsies (SLNB) in our study, including 2 patients with distant metastases (one liver metastases and the other bone and ovary metastases, respectively), 1 with regional lymph node metastases, and 1 with local recurrence and brain metastases.

**Table 5:** Univariate analysis of LR, RR, and DM of young breast cancer patients

Characteristics	LR		RR	DM		
	HR (95% CI)	*P*	HR (95% CI)	*P*	HR (95% CI)	*P*
Tumor size		< 0.01		< 0.01		< 0.01
T1	1.0		1.0		1.0	
T2	0.8 (0.5–1.3)	0.40	0.9 (0.5–1.7)	0.85	1.9 (1.3–2.7)	< 0.01
T3	2.2 (1.1–4.5)	0.02	3.4 (1.6–7.0)	< 0.01	5.7 (3.7–8.7)	< 0.01
T4	8.5 (3.3–22.0)	< 0.01	9.2 (3.1–27.0)	< 0.01	13.7 (7.1–26.5)	< 0.01
Stage		< 0.01		< 0.01		< 0.01
I	1.0		1.0		1.0	
II a	1.5 (0.8–2.9)	0.22	0.4 (0.2–1.1)	0.09	2.1 (1.2–3.5)	< 0.01
II b	2.0 (1.0–4.3)	0.06	2.0 (0.9–4.5)	0.08	3.4 (1.9–6.0)	< 0.01
III a	1.0 (0.4–2.7)	0.92	3.6 (1.7–7.5)	< 0.01	6.2 (3.6–10.6)	< 0.01
III b	7.8 (1.8–34.3)	< 0.01	4.3 (0.6–33.1)	0.16	14.8 (5.5–39.6)	< 0.01
III c	3.8 (1.5–9.5)	< 0.01	4.1 (1.8–9.1)	< 0.01	12.9 (7.6–21.8)	< 0.01
LN metastasis		< 0.01		<0.01		< 0.01
N0	1.0		1.0		1.0	
N1	2.3 (1.4–3.7)	< 0.01	2.3 (1.1–4.6)	0.02	2.1 (1.5–3.0)	< 0.01
N2	1.0 (0.4–2.3)	0.95	5.9 (3.0–11.5)	< 0.01	3.8 (2.6–5.6)	< 0.01
N3	3.9 (2.1–7.3)	< 0.01	6.9 (3.3–14.5)	< 0.01	8.1 (5.6–11.8)	< 0.01
HG		0.09		0.81		0.21
I	1.0		1.0		1.0	
II	0.6 (0.2–1.6)	0.26	0.7 (0.2–2.4)	0.60	1.9 (0.7–5.3)	0.19
III	1.1 (0.4–3.3)	0.86	1.0 (0.3–3.5)	0.97	2.5 (0.9–7.0)	0.08
Final surgery		0.13		0.30		< 0.01
Mastectomy	1.0		1.0		1.0	
BCS	1.4 (0.9–2.3)	0.13	0.7 (0.4–1.3)	0.30	0.4 (0.3–0.7)	< 0.01
ER status		< 0.01		0.16		0.35
Positive	1.0		1.0		1.0	
Negative	2.0 (1.3–3.1)	< 0.01	1.6 (1.0–2.7)	0.06	1.2 (0.9–1.6)	0.25
PR status		< 0.01		0.94		0.93
Positive	1.0		1.0		1.0	
Negative	1.9 (1.2–2.9)	< 0.01	1.1 (0.7–1.9)	0.72	1.1 (0.8–1.4)	0.71
HER2 status		< 0.01		0.59		< 0.01
Positive	1.0		1.0		1.0	
Negative	0.5 (0.3–0.8)	< 0.01	0.8 (0.4–1.4)	0.38	0.6 (0.4–0.8)	< 0.01
MS		< 0.01		0.66		< 0.01
HR+/HER2−	1.0		1.0		1.0	
HR+/HER2+	1.7 (0.8–3.4)	0.15	1.0 (0.4–2.3)	0.94	1.5 (1.0–2.2)	0.06
HR−/HER2+	3.5 (1.8–6.7)	< 0.01	1.7 (0.8–3.9)	0.18	1.9 (1.2–2.9)	< 0.01
HR−/HER2−	1.5 (0.8–2.7)	0.20	0.9 (0.4–1.8)	0.73	0.7 (0.5–1.1)	0.15
NACT		0.04		< 0.01		< 0.01
No	1.0		1.0		1.0	
Yes	1.8 (1.0–3.0)	0.04	2.5 (1.4–4.3)	< 0.01	2.9 (2.1–3.8)	< 0.01
ACT		0.18		0.70		0.29
A- and T-based	1.0		1.0		1.0	
A-based	1.2 (0.7–2.2)	0.47	1.3 (0.7–2.5)	0.48	1.2 (0.8–1.7)	0.42
Radiotherapy		0.20		0.03		< 0.01
No	1.0		1.0		1.0	
Yes	1.1 (0.7–1.8)	0.56	1.9 (1.1–3.3)	0.03	2.5 (1.8–3.5)	< 0.01
ET		< 0.01		0.09		0.22
No	1.0		1.0		1.0	
Yes	0.5 (0.3–0.7)	< 0.01	0.7 (0.4–1.3)	0.30	1.1 (0.8–1.5)	0.71
OFS		0.52		< 0.01		< 0.01
No	1.0		1.0		1.0	
Yes	1.2 (0.6–2.1)	0.64	3.4 (1.8–6.2)	< 0.01	3.7 (2.6–5.3)	< 0.01
TT		0.30		0.94		0.14
No	1.0		1.0		1.0	
Yes	1.5 (0.7–3.4)	0.30	0.9 (0.3–2.6)	0.94	1.4 (0.9–2.3)	0.14

*UA*, univariate analysis; *MA*, multivariate analysis; *HG*, histological grade; *MS*, molecular subtype; *NACT*, neoadjuvant chemotherapy; *ACT*, adjuvant chemotherapy; *ET*, endocrine therapy; *TT*, trastuzumab therapy

**Table 6:** Multivariate analysis of LR, RR, and DM of young breast cancer patients

Characteristics	LR		RR	DM		
	HR (95% CI)	*P*	HR (95% CI)	*P*	HR (95% CI)	*P*
Tumor size		< 0.01		0.11		0.01
T1	1.0		1.0		1.0	
T2	0.5 (0.2–1.0)	0.05	0.6 (0.3–1.4)	0.24	1.4 (0.9–2.3)	0.16
T3	1.7 (0.6– 4.8)	0.33	1.2 (0.5–2.9)	0.74	1.8 (1.0–3.2)	0.04
T4	6.5 (1.2–36.9)	0.03	4.2 (1.0–17.2)	0.05	5.4 (2.0–14.0)	< 0.01
Stage		0.24		0.09		0.57
I	1.0		1.0		1.0	
II a	1.7 (0.7–4.2)	0.22	0.5 (0.2–1.6)	0.26	1.4 (0.7–2.8)	0.28
II b	2.1 (0.6–7.8)	0.25	2.2 (0.6–8.8)	0.25	1.3 (0.5–3.4)	0.53
III a	0.7 (0.2–3.5)	0.69	0.9 (0.2–4.4)	0.90	2.1 (0.8–5.8)	0.15
III b	0.9 (0.1–9.0)	0.96	0.3 (0.0–4.8)	0.43	1.2 (0.3–4.9)	0.81
III c	0.5 (0.1–3.6)	0.53	0.2 (0.0–1.5)	0.11	4.2 (1.0–17.8)	0.05
LN metastasis		0.06		0.02		0.59
N0	1.0		1.0		1.0	
N1	2.0 (1.0–4.0)	0.05	1.6 (0.6–4.1)	0.32	1.5 (0.9–2.6)	0.14
N2	1.01 (0.3–3.5)	0.93	7.1 (1.7–29.2)	< 0.01	1.3 (0.6–2.9)	0.50
N3	6.9 (1.4–35.0)	0.02	34.7 (4.0–304.1)	< 0.01	1.3 (0.3–4.7)	0.73
Final surgery		–		–		0.14
Mastectomy	–		–		1.0	
BCS	–	–	–	–	0.7 (0.4–1.1)	0.14
ER status		0.70		–		–
Positive	1.0		–		–	
Negative	1.5 (0.6–3.9)	0.40	–	–	–	–
PR status		0.54		–		–
Positive	1.0		–		–	
Negative	1.5 (0.6–3.6)	0.41	–	–	–	–
HER2 status		0.92		–		–
Positive	1.0		–		–	
Negative	0.0 (0.0–NA)	0.92	–	–	–	–
MS		0.49		–		0.01
HR+/HER2−	1.0		–		1.0	
HR+/HER2+	0.0 (0.0–NA)	0.92	–	–	1.4 (0.9–2.1)	0.15
HR−/HER2+	0.0 (0.0–NA)	0.92	–	–	2.5 (1.5–4.4)	< 0.01
HR−/HER2−	0.3 (0.1–1.2)	0.10	–	–	1.9 (1.1–3.2)	0.02
NACT		0.57		0.11		0.04
No	1.0		1.0		1.0	
Yes	1.2 (0.7–2.2)	0.57	1.6 (0.9–3.0)	0.11	1.4 (1.0–2.0)	0.04
Radiotherapy		–		0.10		0.64
No	–		1.0		1.0	
Yes	–	–	0.5 (0.2–1.1)	0.09	1.2 (0.7–2.0)	0.41
ET		0.07		–		–
No	1.0		–		–	
Yes	0.4 (0.2–1.0)	0.04	–	–	–	–
OFS		–		0.03		< 0.01
No	–		1.0		1.0	
Yes	–	–	2.4 (1.2–4.6)	< 0.01	3.7 (2.3–5.8)	< 0.01

*UA*, univariate analysis; *MA*, multivariate analysis; *HG*, histological grade; *MS*, molecular subtype; *NACT*, neoadjuvant chemotherapy; *ACT*, adjuvant chemotherapy; *ET*, endocrine therapy; *TT*, trastuzumab therapy; *NA*, not arrived

### Survival outcomes

**Fig. 3:**
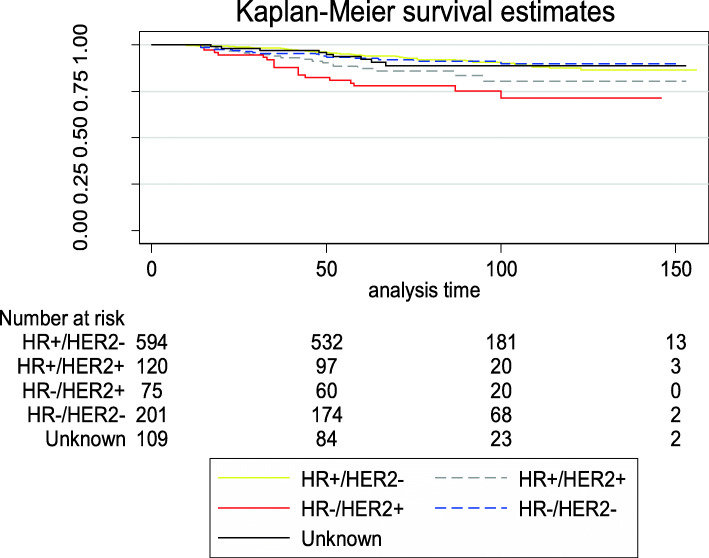
OS of young patients with breast cancer according to various molecular subtypes

**Fig. 4:**
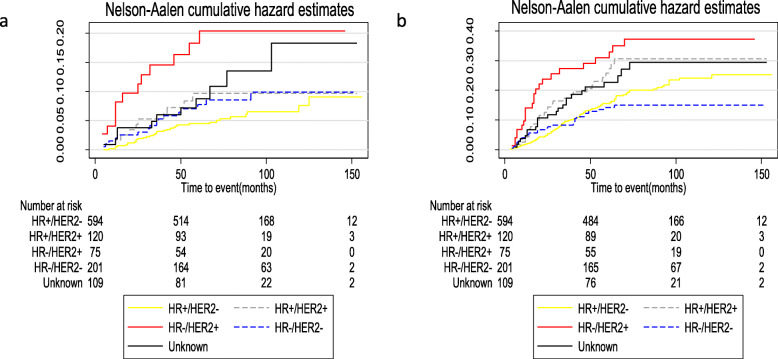
Nelson-Aalen cumulative hazard estimates for LRFI (**a**) and DMFI (**b**) for all young patients by molecular subtypes

HR−/HER2+ patients had the worse OS compared with patients with the other subtypes (*P* < 0.001; Fig. 3). Table 7 lists the relapse and OS for patients with various molecular subtypes. HR−/HER2+ patients had the worst LRFI, RRFI, DMFI, and OS compared with patients with the other subtypes. The median follow-up time was 82 months (range, 5–156 months). In the overall population, the 5-year survival of young patients with breast cancer surgically treated in our institution exceeded 90%. The 5-year OS for patients with HR+/HER2−, HR+/HER2+, HR−/HER2+, and HR−/HER2− was 94.3% (95% CI, 92.0–95.9%), 87.3% (95% CI, 79.5–92.3%), 77.9% (95% CI, 66.5–85.8%), and 92.7% (95% CI, 88.0–95.6%), respectively. The 5-year LRFI and RRFI were highest in patients with the HR+/HER2− subtype (95.6% [95% CI, 94.0–97.0%] and 95.5% [95% CI, 93.4–97.0%], respectively). For patients with triple-negative tumors, the 10-year DMFI was > 85%, which was higher than the other molecular subtypes. Figure 4 shows the Nelson-Aalen cumulative hazard rates for LRFI and DMFI by tumor subtype. Patients with HR−/HER2+ status had a significantly higher LR (HR, 20.4; 95% CI, 11.8–35.4) and DM (HR, 37.2; 95% CI, 24.6–56.3) at 10 years.

**Table 7:** Relapse and survival of outcomes in young breast cancer patients according to various molecular subtypes

Outcome	5-year			10-year		
	5-year estimate (%)	95% CI	Total number of events	10-year estimate (%)	95% CI	Total number of events
Overall						
LRFI	93.3	91.6 to 94.7	61	90.4	87.9 to 92.5	71
RRFI	95.0	93.4 to 96.1	47	92.4	90.1 to 94.2	57
DMFI	83.5	81.1 to 85.6	155	78.3	75.4 to 80.9	186
OS	91.9	90.1 to 93.4	76	86.2	83.2 to 88.7	101
HR+/HER-						
LRFI	95.6	94.0 to 97.0	25	92.7	89.0 to 95.1	32
RRFI	95.5	93.4 to 97.0	25	91.9	88.3 to 94.5	34
DMFI	85.6	82.4 to 88.2	83	78.5	74.4 to 82.1	107
OS	94.3	92.0 to 95.9	32	87.6	83.2 to 91.0	49
HR+/HER+						
LRFI	90.7	83.4 to 94.9	10	90.7	83.4 to 94.9	10
RRFI	94.5	88.0 to 97.5	6	94.5	88.1 to 97.5	6
DMFI	77.1	68.1 to 83.8	26	73.4	63.9 to 80.8	29
OS	87.3	79.5 to 92.3	14	80.3	68.1 to 88.3	17
HR−/HER+						
LRFI	83.1	72.0 to 90.0	12	81.3	69.9 to 88.8	13
RRFI	91.8	82.7 to 96.3	6	90.0	80.0 to 95.1	7
DMFI	71.7	59.9 to 80.5	20	68.6	56.6 to 77.9	23
OS	77.9	66.5 to 85.8	16	71.4	56.8 to 81.8	18
HR−/HER2−						
LRFI	92.5	87.6 to 95.5	14	90.6	84.7 to 94.3	16
RRFI	94.7	90.4 to 97.1	10	94.7	90.4 to 97.1	10
DMFI	86.7	81.1 to 90.8	26	86.1	80.3 to 90.2	27
OS	92.7	88.0 to 95.6	14	89.9	83.8 to 93.8	17

## Discussion

We found a statistically different decreasing trend in the LR and RR rates over time in this large retrospective cohort study of young women with operable invasive breast cancer. This research also revealed that the LR and DM rates varied with the molecular subtype. Tumor size and endocrine therapy were associated with LR, while lymph node metastases and suppression of ovarian function impacted RR based on the multivariate analysis. The 5-year OS of YBC patients was > 90%, with HR−/HER2+ tumors having the worst survival.

The overall 5-year rates of developing LR, RR, and DM were 6.7%, 5.1%, and 16.6%, respectively. Several studies have reported various rates of LRR of YBC patients. LR occurred in 5.4% of the entire population (7.6% of those who underwent breast-conserving surgery [BCS] and 2.6% of those who underwent a mastectomy). An RR of 0.6% after BCS versus 2.6% after mastectomy during 11 years of follow-up in women with breast cancer ≤ 35 of age were collected from the Ontario Cancer Registry between 1994 and 2003 [13]. A study conducted by Aalders et al. reported that young patients < 35 years of age with early-stage breast cancer had a 5-year cumulative incidence of LR, RR, and DM of 3.5%, 3.7%, and 13.9% between 2003 and 2008, respectively [14]. Another study reported a cohort of 3024 patients 18-40 years of age diagnosed with breast cancer a 5-year LRR rate of 2.63% after mastectomy versus 5.33% after BCS (HR, 3.39; 95% CI, 2.03–5.66; *P* < 0.001) [12]. The previous studies likely showed lower rates of LRR because early-stage breast cancer accounted for a large proportion of the study subjects. Patients with stages I and II breast cancer made up 75% of the cohort in our study, while the percentage reached 95% in the study conducted by Aalders et al. [14].

The rates of LR and RR demonstrated a significant decreasing trend during the period of our study. The results of our research were consistent with previous studies [14–16, 21, 35]. A study conducted by Cossetti et al. divided 7178 patients with biopsy-proven stage I-III breast cancer into cohort 1 (C1) and 2 (C2) who were diagnosed between 1986 and 1992, and mid-2004 and 2008, respectively. The authors demonstrated that the hazard rate of relapse was nearly halved in all yearly intervals to year 9 in C2 compared with C1 among the overall population [21]. The patients < 40 years of age in this research accounted for 13.2% of patients, and a subsequent study involving patients < 35 years of age showed overall 5-year rates for LR and RR decreased over time [14]. We studied the time trend of tumor characteristics and treatment modalities and per incidence year of patients. It revealed that the proportion of stages I and II breast cancer increased, while stage III showed a downward trend over the 9 years. The proportion of patients received with anthracycline- and taxane-based chemotherapy regimens and treated with trastuzumab increased during the study time. These findings might explain, in part, the decreasing trend of LR and RR rates over time.

We observed a downtrend in the recurrence of DM over time, although the difference was not statistically significant. Previous studies have reported similar results [14, 17, 36]. Therefore, we suggest that the improvement in OS among patients with breast cancer is closely associated with the lower DM rates in recent years [16, 19, 37, 38].

Our study reported BCS did not significantly affect LRR of young patients with breast cancer. In previous studies, BCS was associated with an increased risk of LR in young patients [39, 40]. Owing to a significant advancement in the treatment of breast ancer, current studies showed BCS had no significant increase in risk of recurrence compared with mastectomy [15, 41].

Patients with HR−/HER2+ tumors (HER2 over-expressing tumors) had the highest LR rates, while HR+/HER2− tumors (luminal tumors) displayed the lowest LR rates among the entire cohort. A systematic review identifying patients from 15 studies appraised the effect of molecular subtype on LRR according to the type of surgery and the authors suggested patients with triple-negative and HER2 over-expressing subtypes were at high risk of developing LRR, and luminal tumors exhibited the lowest LRR rates [25], which was in agreement with our findings. A cohort of 394 early-stage invasive breast cancer patients undergoing BCS were classified as luminal A, luminal B, HER-2, and basal phenotype. The reported crude LRR rates of the basal phenotype were highest (17.3%), followed by HER-2 (15.4%), luminal B (8.7%), and luminal A (5%) [24]. A five-biomarker panel (ER, PR, HRE-2, CK5/6, and EGFR) was used to categorize the tumors, which is not a commonly intrinsic molecular phenotype of breast cancer, and therefore it is not useful clinically. However, the results of our research differed slightly from those of published studies [14, 26, 42]. These studies reported no difference in LR among patients with various tumor subtypes. We found that molecular subtype was a prognostic factor for both LR and DM, but not an independent prognostic factor for LR based on the Cox proportional hazard model.

We found the cumulative probability of 5- and 10-year OS was 91.9% and 86.2%, respectively, in YBC patients < 35 years of age in our study. A population-based study of women diagnosed with breast cancer from 1992 to 2005 demonstrated that the breast cancer-specific survival of patients < 35 years of age was 69% at the 10-year follow-up evaluation [27]. Miller et al. reported that the 5-year breast cancer net survival in females diagnosed between 2001 and 2009 was 88.2% independent of race and age, and the survival rates improved from 2001 and 2003 to 2004 and 2009 [38]. Another study suggested that the 5-year breast cancer-specific survival increased from 74.0% during 1975–1979 to 88.5% during 2010–2015 in women diagnosed between ages 20 and 39 years from the SEER database [20]. The data obtained in our research were slightly higher than previous studies, which might be due to the recent study year accompanied by the improved treatment methods. In addition, the 10 years of follow-up data were not available for patients between 2010 and 2014. Lastly, our study might be limited by the single-center and retrospective nature. In short, the survival rate of YBC patients has improved in recent years.

The characteristics and treatment of BC appeared to be different between young and old women. Mustacchi et.al reported 85.5% of patients aged ≥ 65 years had at least one positive receptor while the proportion in our cohort was 64.9%. The use of chemotherapy (especially taxane regimen) was significantly decreasing with age in old patients [43]. However, almost all the young patients received chemotherapy after surgery and the use of taxanes increased over time in our study. The 5-year rates of LR were higher in young patients (6.7%) than patients ≥50 years (3.7%), whereas the 5-year OS (91.9%) was comparable with OS reported for older patients (91.0%) [44]. Possible explanations are that younger patients might be treated more aggressively after LR and have fewer comorbidities and other diseases than older patients.

Our findings demonstrated that the differences in prognosis among YBC patients varied the with molecular subtype. Women with HR−/HER2+ had the worst LRFI, RRFI, DMFI, and OS compared with the other subtypes, which was consistent with previous articles [24, 29, 30, 33]. Nevertheless, many studies have indicated that YBC patients with luminal B subtype had a worse prognosis [27, 28, 31, 32]. The reason causing the discrepant results might be connected to the year of the study (i.e., there was no HER2-targeted therapy until 1998). After the development of HER2-targeted therapy, the survival of HER2-positive patients was greatly improved [45]. We found HR−/HER2+ had the worst prognosis in our study. It was slightly inconsistent with the current view that triple-negative breast cancer had the worst prognosis. The reasons might be that patients with HR−/HER2+ statuses had larger tumors and more lymph node metastases. In addition, inferior treatment might be another reason causing poor prognosis. Only 83 cases received trastuzumab therapy of the 194 HRE2-positive patients in our study. With the rapid development of HER2-targeted therapies, such as the combination of trastuzumab and pertuzumab, and neratinib and T-DM1, the outcomes of HER2-positive patients could be further improved [46–48].

However, there were some limitations in our study. First, molecular subtypes were categorized according to HR and HER2 status without other marks, such as Ki-67, and analyses of HER2 status were limited by FISH testing that was not performed in some cases. Thus, we could not further subdivide the molecular subtypes. Second, information concerning adherence to adjuvant endocrine therapy and ovary function suppression, such as goserelin, was not available on medical records obtained through the subsequent follow-up. Therefore, the reliability of information might be affected by recall bias. Third, the median follow-up of 82 months was relatively short for YBC patients. Finally, the patients were collected in a large single center in northern China and is not population based. As a result, the experiences of patients in our study might not be generalizable to all young women with breast cancer.

## Conclusions

In conclusion, the overall 5-year LR and RR rates with YBC patients were low and showed a decreasing trend and the proportion of early-stage breast cancer increased between 2006 and 2014. The highest LR rates in this young population were associated with HR−/HER2+ tumors. We expect to develop more new treatments to prolong the survival time and improve the quality of life of young women with breast cancer in the near future.

## Data Availability

The datasets used during the present study are available from the corresponding author on reasonable request.

## Abbreviations

LR: Local recurrenceRR Regional recurrence
LRR: Loco-regional recurrence
YBC: Young breast cancer
HR: Hormone receptor
HER2: Human epidermal growth factor receptor 2
OS: Overall survival
DM: Distant metastasis
LRFI: Local recurrence-free interval
DMFI: Distant metastasis-free interval
RRFI: Regional recurrence-free interval
ER: Estrogen receptor
